# Systematic Review of Penetrating Cardiac Injury by a Firearm: Forensic Implications

**DOI:** 10.3390/healthcare11020265

**Published:** 2023-01-14

**Authors:** Francesco Sessa, Giuseppe Cocimano, Massimiliano Esposito, Pietro Zuccarello, Edmondo Scoto, Pietro Mazzeo, Monica Salerno

**Affiliations:** 1 Department of Medical, Surgical and Advanced Technologies “G.F. Ingrassia”, University of Catania, 95121 Catania, Italy; 2 Department of Mental and Physical Health and Preventive Medicine, University of Campania “Vanvitelli”, 80121 Napoli, Italy

**Keywords:** forensic science, penetrating cardiac injuries, firearms wounds, survival rate, hemodynamic stability

## Abstract

Penetrating injuries of the heart, named penetrating cardiac injury (PCI), may cause hemorrhagic shock as well as cardiac tamponade, leading to death if not treated immediately. This systematic review aims to highlight the main aspects of penetrating cardiac injuries after firearm wounds. The cases of 39 subjects (age 37.05 + 15.4) were selected (6 fatal cases). Specifically, 4/39 cases involved subjects under 18 y.o.; analyzing the entrance wound, in 30/39 cases it was located in the anterior chest, 4/39 in the posterior chest, 3/39 in the shoulder/axilla area, 1/39 in the neck, and 1/39 in the pelvis (gluteus). The exit wound was found in only 3/39 cases. Several factors may influence the prognosis: firstly, prompt intervention represents a crucial point, then considering the complications related to PCI, the most important are myocardial infarction, and projectile migration with embolization. The mortality rate is related to: (1) area and severity of the heart injury; (2) duration of transport and intervention; (3) contemporary lesion to other organ/s; (4) the quantity of blood lost; (5) and presence/absence of cardiac tamponade. Based on these findings, a correct approach in the management of PCI may be considered important from a forensic point of view, both as regards to medical liability and from the trial perspective.

## 1. Introduction

Penetrating cardiac trauma usually generates lethal injuries. Penetrating injuries of the heart, named penetrating cardiac injury (PCI), may cause hemorrhagic shock as well as cardiac tamponade, leading to death if not treated immediately [1,2]. PCIs are traditionally related to stab wounds such as knife injury or firearm wounds. Moreover, penetrating cardiac trauma may be caused by non-firearm-related blast injuries [3].

In the case of firearm wounds, the relative cardiac injuries are particularly severe: about 90% of victims die before arriving at a hospital [4]. On the contrary, the survival rate of the hospitalized cases is estimated between 20–75% [3]. Clinical outcome is related to the condition at first observation at the hospital, ranging from complete hemodynamic stability to cardiac arrest. It is important to note that in the last few years, survival rates have increased in consideration of improvements in prehospital care, diagnostic procedures, and surgical procedures [5].

Victim survival rate is related to different variables such as age, type of injury (it is related to the different ammunitions and relative weapons), physiopathological state, and the association with other organ damage [6].

In this study, we performed a systematic review concerning PCI following firearm wounds, analyzing original articles and case reports with the aim of defining the main aspects, such as the importance of crime scene investigation (CSI), radiological and clinical data, and the role of autopsy in fatal cases.

## 2. Materials and Methods

A systematic review of the literature was performed according to the PRISMA guidelines [7]. The literature review was performed using PubMed and Scopus databases. On these websites, we searched for articles from 1st January 1990 to 1st September 2022 using the following key terms: “Penetrating cardiac injuries” AND “Firearm”, AND “Gunshot”, AND “Weapon”, AND “Gunshot wounds”, AND “Firearm wounds”.

### 2.1. Inclusion and Exclusion Criteria

The case reports and original articles published in peer-reviewed journals were screened if the keywords matched with the “Article title”, “Abstract”, and “Keywords”. To identify further studies that met the inclusion criteria, the references of the selected papers were also reviewed.

The exclusion criteria were: (1) wrong publication type (articles not relevant to the study), (2) review, (3) letters or editorials, (4) articles not in English, (5) meta-analysis, (6) retrospective studies. The inclusion criteria were: (1) original article, (2) case report, (3) articles in English.

### 2.2. Quality Assessment and Data Extraction

M.E. and F.S. analyzed all the articles, evaluating the whole text. In cases of discrepancy of opinions between inclusion or exclusion of articles, they were submitted to M.S.

### 2.3. Characteristics of Eligible Studies

**Figure 1:**
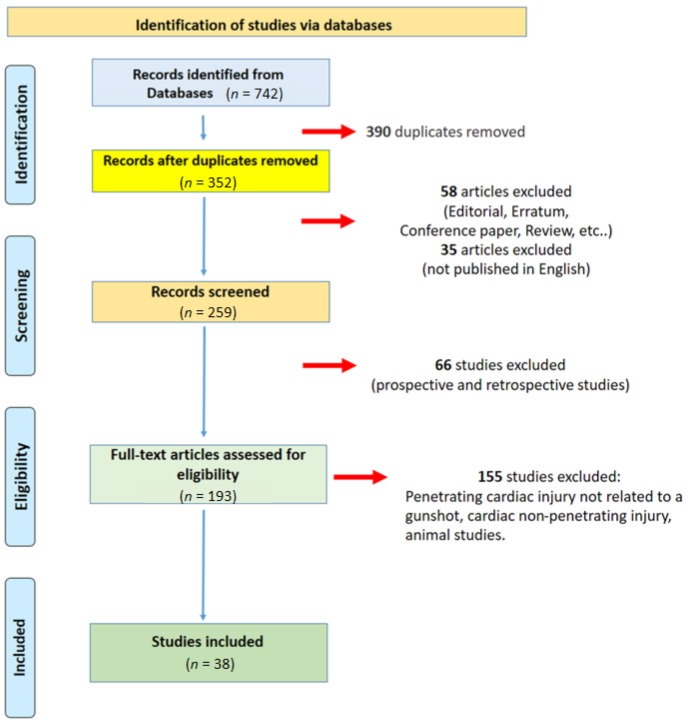
Data source flow chart showing the literature review process and case inclusion. A total of 38 articles were analyzed.

A total of 742 articles were collected. Of these, 390 duplicates were removed. A total of 193 articles were eligible. After an accurate evaluation, 38 articles were included in the present systematic review (Figure 1).

## 3. Results

PCIs are uncommon: it is estimated that less than 10 cases per year were counted in most hospitals [8].

After the literature review, 38 articles (39 cases) were selected, and the main characteristics are summarized in Table 1. The majority of the articles were published in the USA (15), while the other cases belong to the following countries: Brazil (4), Turkey and Germany (3), Italy, France, and Pakistan (2), Nepal, Czech Republic, Spain, Japan, Taiwan, Serbia, Canada, and India (1).

The analyzed cases involved 39 subjects (age 37.05 + 15.4): 33 males (age 37.3 + 16.3) and 6 females (age 35.6 + 9.8).

**Figure 2:**
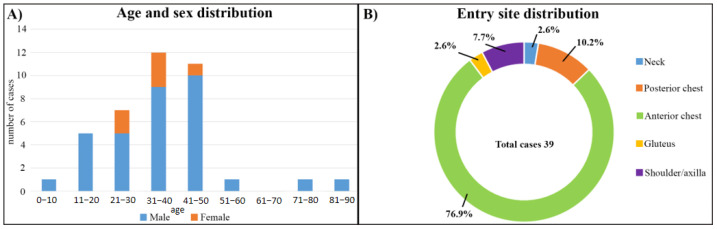
Patients’ demographics and entry site characteristics. (**A**) The age and sex distribution of the affected patients is shown, with most patients being male in the second through fourth decade of life. (**B**) Entry site distribution analysis shows injury to the chest (anterior and posterior), shoulder/axilla region, gluteus, and neck.

As summarized in Figure 2A, 4/39 cases involved subjects under 18 y.o.; despite the risk of death in young children in cases of PCI being higher compared to adults considering the close proximity with other vital organs, in all cases, the victims survived the accident. In particular, Rasool et al. (2014) [27] reported the case of a 10-year-old boy with a PCI and lesion of different organs such as the stomach and liver. Despite these severe clinical conditions, the boy survived surgical intervention. Lovasik et al. (2021) [11] described the case of a young male with a single gunshot wound to the right chest who was discharged 14 days after admission. Knowlin et al. (2018) [13] described the case of a healthy 11-year-old male after sustaining a gunshot wound with an entrance in the left posterior axillary line and exit wound in the anterior chest; despite the severity of the lesions, cardiac function was grossly normal. A prompt recovery was reported by Abou-Leila and Voronov (2017) [17]; in their case, the young victim was discharged 4 days post-intervention.

As summarized in Figure 2B, analyzing the entrance wound, in 30/39 cases, it is located in the anterior chest, 4/39 in the posterior chest, 3/39 in the shoulder/axilla area, 1/39 in the neck, and 1/39 in the pelvis (gluteus). Nevertheless, as reported by Ovali et al. (2021) [9], the PCI may not be related to a thoracic wound, but the injury zone may originate from a gunshot in the neck, as in their case. In the same way, Romero-Velez et al. (2020) [12] presented the case of a victim with multiple gunshot wounds; however, the heart injuries originated from the right gluteal fold, whichmay be considered as an atypical area for PCI.

Analyzing all cases (39/39), the exit wound was found in only 3/39 cases [13,26,36]; a particular case is described by De Giorgio and Raino (2007) [36]: they described multiple gunshots with PCIs, which may be considered fatal for the victim. The peculiarity of this case is represented by the penetration of the two bullets into the heart with a single, common exit wound, demonstrating the importance of the post-mortem examination in similar cases in order to define the exact dynamics. The shot distance does not influence the possibility that the projectile may be retained; Konecny et al. (2016) [23] described an attempted suicide, and despite the short distance, the projectile was retained.

There were six fatal cases (five males and one female), with an average age of 53.6 +19.8, much higher compared to data of all selected cases. Analyzing the scenario of the fatal cases, four were suicides, one was homicide, and another one was not classified by the authors. In three cases (two suicides and one homicide), the subject died at the crime scene; in all these cases, an autopsy was performed, confirming severe cardiac injuries in two cases [14,36], while in another one, the subject shot another bullet to his head after a shot to his thorax [29]. In one case, the woman died immediately after she had arrived at the emergency department [31], while in the other two cases, one patient died for multiorgan failure (the delay in the arrival at the hospital was decisive for the patient’s death) [30], and another from a cerebrovascular event [42]. In the fatal case reported by Cvetkovic et al. (2018) [14], there were two gunshot wounds, hitting the heart at different points. The forensic interest of the discussed case was related to the fact that this was a suicide with multiple gunshots. Usually, in similar cases, there is the suspicion of homicide: following a careful evaluation, the authors justified the two gunshots, confirming the importance of an autopsy.

It is important to note that several factors may influence the prognosis of victims with a PCI: firstly, prompt intervention is a crucial point. Faschingbauer et al. (2006) [38] described a case of a single gunshot with penetrating cardiac injury: the patient survived thanks to prompt surgery. Siddiqui et al. (2015) [25] discussed two cases proposing a different surgical approach in relation to the heart wound. Particularly, in their cases, no exit wound was detected. Both patients survived after prompt medical and surgical interventions. As reported by Tran et al. (2021) [10] and Karigyo et al. (2011) [32], to reduce the risks of cardiac tamponade, prompt surgery is important, particularly in the case of a retained bullet. The case reported by Incorvaia et al. (2007) [37] is singular, with the victim hit in the chest by a bullet that fell from the sky. Despite the bullet falling at terminal velocity compared to their initial muzzle velocity, it was sufficient to cause significant injuries. In the described case, the bullet was able to injure four organs, including the heart, diaphragm, stomach, and spleen. Prompt surgery may be decisive in cases of severe injuries, such as the presence of a hole in both ventricles, such as in the case reported by Kwan et al. (1995) [44]: with prompt surgery the victim survived the accident.

The most important complications related to PCI are myocardial infarction [40] and projectile migration with embolization [20]. Particularly, myocardial infarction could be related to the abuse of different substances; however, in the analyzed cases, the toxicological investigations were reported in only two cases [33,36], while this tool may be considered fundamental in order to ascertain the exact cause of death [47,48,49,50,51].

Considering the complications that may be related to the PCI, Obrador et al. (2015) [24], in their case report, discussed the risk of infection developing, such as pneumonia in victims with PCI. In particular, the authors strongly encouraged post-operative follow up in order to reduce these risks. Ustin et al. (2011) [33] reported the case of a pregnant woman who attempted suicide. Despite the woman surviving the attempted suicide, and the baby being delivered at term, the subsequent clinical investigation demonstrated pituitary infarction after hemorrhagic shock, emphasizing the importance of considering endocrine dysfunction in all cases of persistent hypotension. In the evaluation of the severity of the injury, as reported by Rupprecht and Gaab (2018) [15], the rib may result decisive in reducing the severity of heart lesions.

Based on the selected cases, it is possible to have adverse effects or complications after several hours or days post-accident. Sapkota and Koirala (2016) [21] presented a case of a singular wound to the heart, with the patient admitted to the emergency room after 9 h; nevertheless, the victim survived the operation, returning to his normal life. Volpe et al. (2018) [16] discussed a singular case of a victim with two retained projectiles, with a penetrating cardiac injury. In this case, the symptoms started 16 days after the accident; the location near the apex of the right ventricle could be considered decisive in the containment of the bleeding. Nevertheless, the subject was operated on and discharged. Similarly, Meira et al. (2005) [39] discussed the case of a subject with a penetrating injury and a stable hemodynamic condition: surgery to remove the bullet from the heart was performed 11 days after the accident.

Another important consideration is related to the opportunity to perform a radiological investigation, particularly when the projectile is retained. The chest-X ray represents the basic approach: it should be considered mandatory, and it is usually supported by CT, ECHO, and FAST. As highlighted by different authors [11,17,18,22,26,28,41,42,43,45,46], the support of radiographic investigation, as well as the CT scan, may be considered fundamental in order to identify the retained projectile, guiding surgical operations. This concept is reported by Fu et al. (2017) [19], particularly when multiple lesions on the heart were detected. In only 1/39 cases [12], did the authors report that the victim was operated on without radiological investigation because of the severity of the lesions. The role of radiological support is important in the evaluation of projectile trajectory in order to evaluate the possibility of other organ damage. As previously described, the possibility to involve other abdominal organs is possible in young subjects as well as in adults, as reported by Harter et al. (2010) [34] and Ellertson and Johnson (2008) [35].

## 4. Discussion

According to the literature, there are several important considerations concerning PCI after a gunshot. PCIs were first described by Hollerius in 1868 [52]. Moreover, in 1989, Noughton et al. suggested that timely transport, resuscitation, and immediate surgery represent the main essential aspects in the management of penetrating cardiac trauma [53]. A first confirmation was given by Campbell et al. in 1997. They analyzed 1198 PCI cases in South Africa, finding that about 6% reached the hospital alive, while 94% were transferred directly to the mortuary [54]. Mittal et al. analyzed the outcome of patients with PCI in a level II Trauma Center in northern Detroit for 14 years: 45% of victims reported gunshot wounds and 55% had stab wounds; survival rate was 47% in gunshot injuries and 80% in stab injuries. In this study, the authors confirmed that the mortality rate may be significantly reduced with early diagnosis, rapid transportation to the hospital, and correct treatment [55].

In 1998, Asensio et al. analyzed the survival rate after PCIs, resulting in an overall survival rate of 36.6% [56]. Moreover, the same research group published another report estimating the survival rate at about 33%. In this study, they reported that the severity of hemorrhages and blood pressure were the most relevant factors influencing survival rate [57,58].

Tyburski et al. conducted a retrospective study reporting a survival rate of 23% after a PCIs related to gunshots. Several factors influence the prognosis, such as physiological status at admission, presence of cardiac tamponade, mechanism of injury, and type and number of cardiac chambers involved (survival rate of 51% in single-chamber injuries versus a 13% survival rate in multiple-chamber and great vessel injuries) [59]. In other studies, it has highlighted that the survival rate may be related both to the kind of lesion and to the number of heart chambers involved [60]. With reference to the anatomic site affected, Karaca et al. in 2015 found 17% mortality in cases of exclusive chest involvement, while the worst outcome was observed in cases of multi-chambers or concomitant involvement of other organs [4]. Other authors highlighted the significance of response time and surgical intervention as essential to hemodynamic stability and survival rate [61,62].

Swaroop et al. emphasized the importance of the interval time between accident and rescue operation, demonstrating that longer interval times are strictly related to higher mortality rates, particularly for interval times of 46–60 min. [63]. In 2016, Meizoso et al. confirmed the positive impact in the survival rate of surgical timing: patients having surgery after 10 min had higher mortality compared with those operated on before [64]. In agreement with these findings, Campbell reported that victims who had surgery within 30 min had a better survival rate compared with those in whom surgery was delayed [54]. It is important to note that short interval times are not frequent in real cases.

Other studies have compared the survival rate of stab wound cardiac injuries to the survival rate of gunshot PCIs, concluding that the gunshot injuries resulted in worse outcomes (survival rates were 5%-11.5% for gunshot and 33–50.3% for stab wounds) [65,66,67].

A study performed in a Scandinavian trauma center reported that victims admitted with a gunshot PCI had a survival rate of 50% [68], while a retrospective study conducted in a Brazilian trauma center highlighted that PCI after a firearm incident had a mortality rate of 52.2% [69]. Other studies reported a mortality rate ranging from 39 to 94 % for PCI due to a gunshot/stab wound [70,71,72,73,74,75,76,77].

Tavares Pereira et al. estimated the incidence of penetrating injuries during a 20-year period, comparing the periods 1990–1999 (group 1) and 2000–2009 (group 2); 48.4% of patients sustained stab wounds, and 51.6% were victims of gunshot wounds. The total mortality rate was 16.1%, but the authors observed a trend in mortality reduction when comparing group 1 with group 2 (20.3% versus 10.3%), demonstrating a decrease in mortality over the years [78].

From a forensic point of view, firearm wounds represent a central issue in forensic pathology, particularly in the definition of dynamics and the responsibility on the crime scene, as well as the possible medical doctor responsibility in patient management. In particular, atypical wound site or unexpected trajectories of pellets or bullets may complicate the interpretation of wounds, injuries, and mechanisms, and the post-mortem investigation combined to a multidisciplinary approach became an indispensable tool [79,80]. For example, the use of histological, immunohistochemical, and molecular techniques could be very useful to assess wound vitality and to improve the methods to define the time since death [81,82]. At the external examination of the corpse, forensic pathologists may find either the same number of entrance and exit wounds, or alternatively, a great number of entrance wounds (in the case of retained bullets), or rarely, a great number of exit wounds (in the case of fragmentation or explosion of bullets inside the body). As reported in this review, this last possibility rarely occurred. Moreover, as documented in this literature review, the site of injuries may not be considered fundamental for the survival rate of the victim because PCI may be generated from an atypical injury site (for example from the neck or gluteo). Compared to the site, it is more important the establish the exact trajectory of the projectile.

The most important finding in fatal cases is cardiac tamponade: it is life-threatening, slow, or rapid compression of the heart due to the pericardial accumulation of fluid, pus, blood, clots, or gas, because of effusion, trauma, or heart rupture.

Another crucial point is bullet embolization into the systemic circulation after gunshot injury, particularly when a PCI occurs: it may be fatal for the victim.

In a forensic context, crime scene investigations (CSI) following firearm accidents are also essential for both medical and forensic pathologists in cases of critical and fatal injuries [80]. Important elements such as bullet trajectory can be deduced through CSI and can aid in investigations and autopsies. In this regard, Bonsignore et al. [29] were able to define which shot hit the victim in the heart among different lesions combining the evidence collected during CSI with the autoptic findings. Moreover, understanding the dynamics of the accident is vital for predicting possible organ damage. It is also essential to check the compatibility of the weapon allegedly used with the injury in cases of homicide.

As summarized in our data, the exit wound was reported in only three cases. In our opinion, these findings are strictly related to the fact that a great number of the published papers are related to the surgical techniques to repair heart damage or to remove the retained bullet.

In light of the findings of this review, the mortality rate after a PCI is related to:

√ Area and severity of the heart injury;
√ Duration of transport and intervention;
√ Contemporary lesion to other organ/s;
√ The quantity of blood lost;
√ Presence/absence of cardiac tamponade.

Usually, the first hour after the accident may be considered crucial. A timely diagnosis and subsequently prompt treatment may be decisive to improve the survival rate for the victim, reaching more than 70% [9]. Moreover, after a PCI with subsequent treatment and discharge, the survival rate is about 88% at nine years, although it is important to start a long-term follow-up [10]. Contrariwise, in the absence of treatment in the first hour, the mortality rate is about 75% [9]. Other factors that could positively or negatively influence the patient’s outcome are related to different situations. Indeed, considering that PCI management is a complex challenge, limited experience in the field of routine cardiac surgery, the misrecognition or failure to ascertain concomitant injuries to the chest or abdominal cavity due to the high kinetic energy of the projectile, or secondary injuries due to bone fragments, may be considered negative factors. On the contrary, the arrival of the victim at a top-level trauma center experienced in thoracic cardiac surgery as well as recourse to the multidisciplinary management of complications may be considered positive factors [83,84,85].

Radiological investigations are a very useful tool to identify the retained bullet. Indeed, when there is no exit wound, the retained projectile may generate additional complications, such as infection, ischemia, and thrombosis: each of these conditions could be fatal for the victim. For these reasons, prompt removal should be evaluated in each case. As emerged from the literature review, in these cases, the final location of the projectile is influenced by different factors, such as the entry site, the size and weight of the projectile, the firing dynamic, and the victim’s position after firing, the hemodynamic situation, respiratory motion, and finally the severity of the injury. Based on the discussed data, when the victim is hemodynamically stable, it is strongly recommended to immediately carry out a whole-body radiographic examination, even if the wounds are not located in the chest or abdominal region. The chest X-ray is the fastest screening method, but it should be supported by other radiologic techniques in order to investigate organ injury and the possible bullet embolization pathway. In this way, angiography could be very useful to confirm arterial injury and embolization, while a CT scan may help in the identification of other injuries in vascular structures and organs; ECHO represents a valuable tool to ascertain cardiac damage, particularly in the presence of valvular regurgitation and hemopericardium. It is important to note that the use of radiological techniques is fundamental in the post-mortem examination to establish the trajectory of the bullet, as well as the presence of organ injuries to define the exact cause of death. Moreover, radiological technologies are considered a very useful tool to clarify several important forensic aspects of PCI, as well as to perform a differential diagnosis between entry and exit wounds and estimate the firing distance [84,85,86,87].

This literature review contains several strengths: the use of different keywords, with inclusion and exclusion criteria; the wide temporal period analyzed (from 1 January 1990 to 1 September 2022). At the same time, several limitations are present: the inclusion of articles published only on Pubmed or Scopus; the impossibility of applying a complete statistical analysis due to the small sample size; the absence of different information, such as the presence of pre-existence pathologies, the dynamic of the event, and multisystem lesion involvement. Finally, the toxicological findings were reported in only two articles, while this tool could be considered fundamental in the definition of the exact cause of death.

## 5. Conclusions

In conclusion, the management and prognosis of victims affected by PCIs after a firearm wound relies on several crucial factors. The most crucial factor is the hemodynamic situation after the accident; this is related to the type of firearm lesion. An injury to the heart chambers or vessel lesions can lead to cardiac tamponade, therefore requiring immediate hospitalization, stabilization, and surgery. When the victim is hemodynamically stable in the emergency room, about 60–70% will not need surgery.

When analyzing forensic cases, it is essential to consider the above outcomes: a correct approach in the management of PCI may be considered important from a forensic point of view, both as regards to medical liability and from the trial perspective.

Based on these considerations, the post-mortem examination with a multidisciplinary approach remains the gold standard method in cases of firearm murder, and it should always be performed, even if the dynamics seem clear.

## Figures and Tables

**Table 1:** The main findings of each analyzed case are listed ranking them from the more recent to the oldest.

Authors, Country, and Year	Sex/Age	Entrance Wound	Exit Wound	Heart Injuries	Hemodynamic Stability	Patient Evaluation	Additional Features/Toxicological Investigations	Survival
Ovali et al., Turkey (2021) [9]	Male (M), 46 years old (y.o.)	Gunshot with a bullet wound on his left cervical area (dimension: 1 cm × 1 cm)	No exit wound	Gunshot bullet lodged in the left ventricle (LV).	Stable hemodynamic condition. No cardiac tamponade	Ultrasonography; Computer tomography (CT); Transthoracic echocardiography (ECHO)	Not available (na)/na	Survivor
Tran et al., USA (2021) [10]	M, 34 y.o.	Gunshot wound to the left upper back, just below the left scapula	No exit wound	The bullet entered the posterior wall of the left atrium (LA).	No signs of shock, impending arrest, or cardiac tamponade	Chest X ray; CT scan; Abdominal ultrasound	The victim was discharged 10 days after admission/na.	Survivor
Lovasik et al., USA (2021) [11]	M, 15 y.o.	Single gunshot wound to right chest, just inferior to the mid-clavicle	No exit wound	Wound on the right atrial (RA)	Cardiac tamponade	Ultrasound of the heart; Chest X ray	The victim was discharged 14 days after admission/na.	Survivor
Romero-Velez et al., USA (2020) [12]	M, 30 y.o.	Multiple gunshot wounds. For heart injuries, the bullet had penetrated from the right gluteal fold and traversed the entire torso ending up lodged in the left scapula.	No exit wound	Two large injuries to the LV were reported. The bullet appeared to have entered the LV posteriorly and exited anteriorly.	Cardiac tamponade	Because from the severity of the wound, the victim was promptly operated.	The victim experienced a prolonged hospital recovery/na.	Survivor
Knowlin et al., USA (2018) [13]	M, 11 y.o.	Single gunshot wound close to the left posterior axillary line	Exit wound in the anterior chest just inferior and medial to the left nipple	The pericardium had a small hole from which blood came out under high pressure. Upon opening the pericardium to expose the injury adequately, a hole in the apex of the LV was identified.	Cardiac function was grossly normal	Focused abdominal sonogram from trauma (FAST); Chest X Ray	na/na	Survivor
Cvetkovic et al., Serbia (2018) [14]	M., 34 y.o.	Two gunshot entrance wounds in the left side of the chest	The bullets were found lodged under the skin of the back.	One bullet had passed through the left edge of the sternum, the pericardial sac, the anterior wall of the RV, the interventricular septum, the posterior wall of the LV, the rear aspect of the pericardial sac. The second bullet had passed through the fourth left costal cartilage, the pericardial sac, the upper anterior part of the interventricular septum, the anterior cusp of the mitral valve, the posterolateral wall of the left ventricle, and the pericardial sac.	Massive cardiac hemorrhage	Autopsy	Suicide/na	Deceased
Rupprecht and Gaab, Germany (2018) [15]	M., 49 y.o.	Single gunshot wound 2 cm below the left mammilla	1 cm exit wound on the left-back	A 3 cm contusion lesion with a small bleeding perforation (3–4 mm) in the LV (dorsum of the ramus interventricularis anterior to the left coronary artery) was detected.	After the accident, there was a progressive instability of the victim’s vital signs, he was immediately transferred to surgery.	Sonography of the abdomen (FAST); CT scan	The bullet struck a rib, reducing its energy/na.	Survivor
Volpe et al., Brazil (2018) [16]	M, 34 y.o.	Two projectiles hit the subject in the thorax area, lateral side (subaxillary), at the height of the right hemithorax.	No exit wounds	One projectile was located inside the right ventricle (RV).	The critical condition started 16 days after the accident.	Chest X ray; CT scan; ECHO	The other projectile was located in the right rectus abdominis/na.	Survivor
Abou-Leila and Voronov, USA (2017) [17]	M., 17 y.o.	Single gunshot wound to the left costal margin	No exit wound	CT of the chest and abdomen showed retained foreign body in the interventricular septum.	Stable	Chest X ray; CT scan; ECHO	The victim was discharged on the 4th postoperative day/na.	Survivor
Santos et al., Brazil (2017) [18]	M, 26 y.o.	Single injury to the right hemithorax	No exit wound	CT showed an image suggestive of a projectile fragment in the intracardiac position, located in the membranous septum region, and close to the septal cusp of the tricuspid valve.	Stable	CT scan with and without contrast; ECHO	The victim was discharged on the 4th postoperative day/na.	Survivor
Fu et al., Taiwan (2017) [19]	M., 37 y.o.	One gunshot wound in the anterior chest wall	No exit wound	A penetrating hole identified in the RV wall; another penetrating hole was detected at the tricuspid septal leaflet, extending to the tricuspid annulus.	Stable	Chest X ray; CT scan; ECHO	Multiple lesions on heart (LV, LA, tricuspid leaflet)/na	Survivor
Imbert et al., France (2016) [20]	M, 59 y.o.	Penetrating gunshot wound to the left side of the chest caused by a rifle bullet a. An entrance wound was noted on the left side of the chest, parasternal, next to the fifth intercostal space.	No exit wound	Gunshot wound of the heart with migration of the projectile to the pulmonary artery are rare.	Non-Stable	Chest X ray; CT scan	He was admitted immediately after the accident. He was discharged 22 days after the first surgery/na.	Survivor
Sapkota and Koirala, Nepal (2016) [21]	M, 32 y.o.	The entry wound pierced the sternum laterally at the third intercostal level.	No exit wound.	The first cardiac perforation was seen near the RV outflow tract. The second perforation was at the inferior wall of the LV, 3 cm off the apex.	Stable	Chest X ray; CT scan; ECHO	He was admitted 9 h post shot. He was discharged 14 days after surgery/na.	Survivor
Kaya et al., Turkey (2016) [22]	M., 32 y.o.	A superficial wound on the left shoulder and another on the left side of the thorax at the 6th intercostal space	No exit wounds	A bullet in the pericardial sac with pericardial effusion compressing the heart was found.	Stable (discharged after 7 days)	CT scan;	The gunshot wound was noticed 1 day later/na.	Survivor
Konecny et al., Czech Republic (2016) [23]	Female (F), 39 y.o.	A gunshot wound in the lower third of the sternum	No exit wound	The projectile trajectory fractured the lower third of the chest bone, punctured the anterior walls of the RV and RA, and perforated the inferior vena cava.	Stable (discharged after 6 days). The projectile was found at the level of the right 9th rib paravertebrally.	CT scan	Attempted suicide, psychiatric treatment for two weeks/na.	Survivor
Obrador et al., Spain (2015) [24]	M, 43 y.o.	A wound below the left clavicle	No exit wound	Perforation of the RV and the RA.	Stable	Chest X ray	He was discharged on the 12th postoperative day/na.	Survivor
Siddiqui et al., Pakistan (2015) [25]	M., 48 y.o.	An entry wound medial to the left nipple, close to the midline on the left side	No exit wound	CT scan found hemopericardium and metallic bullet posterior to the heart.	Stable	CT scan	na/na	Survivor
	M., 35 y.o.	A gunshot injury to his anterior chest	No exit wound	RV bullet wound anteriorly, and a LV wound posteriorly.	Cardiopulmonary bypass (CPB) was established. Both ventricular wounds and VSD repaired.	CT scan	na/na	Survivor
Suzuki et al., Japan (2014) [26]	M., 47 y.o.	Single gunshot wound on the left anterior chest wall	15 mm wound on the left posterior wall	The bullet damaged the pericardium and the heart from the apex to a portion of the left ventricular lateral wall.	Unstable	CT scan	Suicide attempt. The patient was discharged on the 18th postoperative day/na.	Survivor
Rasool et al., Pakistan (2014) [27]	M., 10 y.o.	A single gunshot entry wound in the region of the sternum at the level of the 5th intercostal space, with history of stray bullet injury	No exit wound	The cardiac damage resulting from the projectile observed in the anterior wall (entrance hole) and the inferior wall (exit hole) of the RV.	The patient was hemodynamically stable. Different organs were damaged (liver, stomach).	Chest X ray; CT scan	He was discharged on the 9th postoperative day/na.	Survivor
Mills et al., USA (2014) [28]	M., 20 y.o.	Intrathoracic gunshot	No exit wound	There was an entry wound to the left posterior ventricle approximately 1 cm from the atrioventricular groove. The left atrium opened, and the bullet was identified buried beneath the endocardium of the back left atrial wall.	Hemodynamically stable	Chest X ray; CT scan; ECHO	Medical history significant for schizophrenia. He was discharged after 3 weeks, but he was treated for other problems/na.	Survivor
Bonsignore et al., Italy (2013) [29]	M., 75 y.o.	One wound in the left chest in the second infracostal space	No exit wound	The bullet passed through the pericardium, left auricle, puncturing the anterior wall of the left coronary artery.	Hemopericardium	Autopsy	Suicide. After a thoracic wound, he shot another bullet to his head/na.	Deceased
Porcu et al., France (2012) [30]	M., 82 y.o.	One wound in the sub-mammary region, in the left 7th intercostal space	No exit wound	RV injury	Unstable	Chest X ray	He arrived at the emergency department 3 h after attempting suicide/na.	Subject died after 5 days from a multiorgan failure due to a prolonged preoperative low cardiac output.
Branch and Adams, USA (2012) [31]	F., 40 y.o.	A single entrance to the upper-left chest	No exit wound	The LV was lacerated secondary to blast forces, with resultant hemopericardium and subsequent cardiac tamponade.	Unstable	FAST	Suicide/na	Deceased
Karigyo et al., Brazil (2011) [32]	M, 40 y.o.	Left hemithorax	No exit wound	The cardiac injuries were observed in the anterior wall of the LV (inlet hole), and in the posterior region of the RV (outlet hole).	Stable	Chest X ray; CT scan	Victim of an attempted robbery; he was discharged 26 days after the operation/na.	Survivor
Ustin et al., USA (2011) [33]	F., 39 y.o.(pregnant)	A single gunshot wound to the right chest over the third intercostal space (approximately 5 cm right of the sternum)	No exit wound.	The bullet had fractured the sternum and entered the pericardial sac lacerating the myocardium just inferior to the atrioventricular groove.	Unstable	Chest X ray	Attempted suicide in a patient with a past medical history of substance abuse, hepatitis C, and gestational diabetes; Discharged on post-trauma day 17/toxicology screen was positive for cocaine and opioids.	Survivor; the baby was delivered at term
Hartert et al., Germany (2010) [34]	M, 57 y.o.	A single entrance wound in the left mid-paramanubrial area.	No exit wound	The bullet penetrated the anterior wall of the RV causing a pericardial tamponade. The exit hole was located in the posterior area of the RV.	Unstable	Chest X ray	Attempted suicide. The subject was discharged on the 28th postoperative day/na.	
Ellertson and Johnson, USA (2008) [35]	F., 48 y.o.	One gunshot wound entered the left anterior chest wall and appeared to cross the midline inferiorly to rest near the dome of her liver.	No exit wound	The inspection of the heart showed a relatively large (approximately 3 cm long) tangential injury to the RA near the right atrioventricular groove adjacent to the right coronary artery.	Unstable	Chest X ray; CT scan	Two months later, psychiatric issues related to the incident were recorded/na.	Survivor
De Giorgio and Raino, Italy (2007) [36]	M., 45 y.o.	4 penetrating gunshot wounds and one tangential wound. 2 of the penetrating injuries in the chest: the upper bullet had entered the thoracic cavity between ribs III and IV, 3 cm to the right of the edge of the sternum, and the lower bullet entered between ribs IV and V, close to the sternum.	Only one oval exit wound on the posterior wall of the thorax between ribs VII and VIII, 3 cm to the right of the vertebral column.	Both bullets had made entrance wounds on the pericardial sac and the anterior wall of the RA. In the upper posterior atrial wall as well as in the posterior wall of the pericardium, only one wound was visible.	Died at the scene immediately after being shot.	Autopsy	Shotgun homicide/Alcohol use was considered a factor contributing to the death.	Deceased
Incorvaia et al., USA (2007) [37]	M., 47 y.o.	There was a bullet hole in his chest on the left side above the nipple line, in the fifth intercostal space, 2 cm to the left of the sternal edge.	No exit wound	Two holes were exposed in the right ventricle.	Unstable	FAST;	He was struck in the chest by a bullet that fell from the sky. Discharged 30 days postoperatively/na.	Survivor
Faschingbauer et al., Germany (2006) [38]	M., 43 y.o.	An entry wound was observed on the ventral chest just a few centimeters left to the midline at about the fourth rib.	No exit wound	2 cm diameter rough-edged defect in the RA was found. The exit wound at the dorsal border of the superior vena cava to RA.	Unstable	Chest X ray;	Homicide attempt. After that, he was able to run 19 m before collapsing. The victim was discharged about 14 days later/na.	Survivor
Meira et al., Brazil (2005) [39]	M., 29 y.o.	The bullet passed through right arm and penetrated the thorax.	No exit wound	The bullet was lodged in the RV anterior wall.	Stable	Chest X ray	After the 11th day, he had a thoracotomy exploration with subsequent surgery/na.	Survivor
Bali et al., India (2003) [40]	M, 30 y.o.	Right upper anterior chest wall	No exit wound	Perforation of the RV outflow tract.	Stable	Chest X ray	Multiple bullet injuries in the chest and face after being shot. The victim was discharged after 2 days/na.	Survivor
Habdank and Nolan, Canada (2003) [41]	M, 33 y.o.	A single gunshot wound that initially penetrated his left arm and then entered the posterolateral mid left thorax.	No exit wound	Perforation of free wall of the LV.	Stable	Chest X ray; ECHO	The patient was discharged after 7 days/na.	Survivor
Kurt et al., Turkey (2001) [42]	M., 46 y.o.	Entry wound in the right chest at the second intercostal space on the midaxillary line.	No exit wound	Entrance wound in the LA.	Unstable	Chest X ray	The victim died 3 days after the operation for the cerebrovascular event/na.	Deceased
Doty et al., USA (1999) [43]	F., 27 y.o.	Single gunshot wound to the lateral left side of the chest (left fifth intercostal space).	No exit wound	Two cardiac perforations were noted, one in the LA and one in the LV.	Unstable	Chest X ray	Several complications after surgery/na.	Survivor
Kwan et al., USA (1995) [44]	M, 19 y.	Single gunshot wound to the chest	No exit wound	The bullet entered the RV and exited through the LV.	Stable	Chest X ray; ECHO	Dyspnea after surgery/na.	Survivor
Wait et al., USA (1994) [45]	M., 26 y.	Two close-range pistol gunshot wounds to the left chest	No exit wound	One bullet remained in the left upper lung field and was associated with massive hemothorax; another one transversed the mediastinum, entering the obtuse margin of the heart, and proceeding into the right upper quadrant of the abdomen.	Cardiopulmonary resuscitation	Chest X ray; CT scan	Several complications/na.	Survivor
Skipper and Debski, USA (1990) [46]	F., 21 y.	Shotgun blast to left chest and arm.	No exit wound	The pellet holes that were no longer bleeding were found in the myocardium of the LV.	Stable	Chest X ray; Arteriogram	Several complications/na.	Survivor

## Data Availability

The authors confirm that the data supporting the findings of this study are available within the article.
